# Clinical Society of the New York Polyclinic Medical School and Hospital

**Published:** 1905-04

**Authors:** D. S. Dougherty

**Affiliations:** The President; In the Chair


### SOCIETY REPORTS.

### CLINICAL SOCIETY OF THE NEW YORK POLYCLINIC MEDICAL SCHOOL AND HOSPITAL.

Stated Meeting, held March 6, 1905.
The President, Dr. D. S. Dougherty, in the Chair.

### SPECIMEN OF MYOMATOUS UTERUS.

This specimen was exhibited by Dr. L. J. Ladinski, who said he had removed it from a patient twenty-eight years of age, with the following history: Married; began to menstruate when thirteen years old. Had two children, both living and healthy, and had had no abortions. Menstruation of late was very profuse, lasting from ten to fourteen days, and so much blood had been lost that the patient had become very pale and anemic. About a year ago she noticed a lump in her right side, which seemed to diminish in size during the menstrual period, and to increase between these periods. Lately the bowels have not moved freely, and cathartics have been resorted to. For the past three months the tumor increased in size very rapidly, until it reached the umbilicus. There was constipation and constant and painful urination.

Bimanual examination revealed a large tumor filling the pelvic and abdominal cavities and reaching to umbilicus; it was immovable, owing to its fixed position on the right side and in front of the pubes, and appeared to compress the bladder. The tumor involved the entire uterus, and was very soft and boggy to the touch, so much so that it could readily be mistaken for a uterus pregnant between the fifth and sixth month.

A diagnosis of soft myoma was made and operation advised.

Abdominal hysterectomy was done under ether anesthesia two days ago. The ovary and tube of the left side were not removed.

On incising the tumor, it was found to contain a large quantity of calloid material, surrounding several small fibro-myomata, showing in all probability a myxomatous degeneration.

The case is interesting because of the colloid or myxomatous degeneration of the fibroid, a condition which, though not rare, is sufficiently infrequent to warrant reporting, and especially from a diagnostic standpoint.

Soft myomata of the uterus are extremely difficult to differentiate from pregnancy, especially when there is amenorrhea, as frequently happens in these cases. In myomata the uterus is uniformly enlarged and presents on palpation a soft, baggy feeling, simulated very closely by that of pregnancy, and when there are a number of small fibroids imbedded in the tumor, as is sometimes the case, they may be mistaken for fetal parts. Careful palpation will show, however, that in pregnancy the uterus is more uniform in contour and that there is also the characteristic feeling of elasticity and fluctuation to distinguish pregnancy from the soft, boggy, almost mushy feeling of a myoma. The speaker knew of no condition which simulated pregnancy so closely, and which was at times so difficult to distinguish from it. The specimen was to be submitted to microscopical examination in order to determine the exact nature of the tumor and the character of the degenerative changes.

The patient has run a perfectly normal course up to date, and there is every reason to believe that she will have an uninterrupted convalescence.

Dr. F. M. Jeffries said that the colloid degeneration never took place in any tissue other than epithelial, and as the specimen under discussion was a fibroma, he thought the colloid degeneration could be excluded. He also could not recall having heard of a single instance of myxomatous degeneration in a fibroma. From the macroscopical picture presented, he was inclined to think that the change was probably edematous. There are two types of fluctuating fibroma, one in which there is merely a necrosis or breaking down of the cellular structure, and the other simple edema, where the interstices are filled with fluid which may even be partly viscous in character. Of course, to make a positive diagnosis, a microscopical examination would be necessary, but the speaker's diagnosis, from the gross, was an edematous rather than a colloid or a myxomatous degeneration.

A CASE FOR DIAGNOSIS.

Dr. D. A. Sinclair presented a case for diagnosis of poarthortis of a chronic nature. Mr. H., twenty-three years of age, railroad employee by occupation. Mother and father living. Ten or eleven brothers and sisters died in infancy—cause unknown. Family history negative to tuberculosis. Six years ago had an attack of gonorrhea, for which he was treated, and he says was apparently cured. About a year later there was a recurrence of the discharge, lasting about three weeks, there being no apparent cause for this attack. Attacks have been frequent since that time, without the patient having exposed himself to them, all showing an uncured condition since 1899. Fifteen months ago he developed a swelling of the left elbow. He then noticed a pain in the right knee, and then in the left hip; three months later this left elbow opened spontaneously and pus escaped. He stated that during all the time the left elbow was swelling he felt absolutely no pain. At about this time the left index finger, second joint, and the middle finger, at the third or distal joint, swelled, and later the same condition of swelling obtained in the joints of the great and second toes, all rupturing and discharging pus.

The patient entered the hospital January 5th with an acute epididymitis on the left side. This inflammation was accompanied by pain. Hot flaxseed poultices were applied every hour until fluctuation was felt. The abscess was opened under ether and a foul, cheeselike substance was taken away, which had destroyed the epididymis completely.

When he entered the hospital, the patient had a large abscess on the right side, extending from just below the margin of the nipple downward six or seven inches, and about three and a half inches wide, burrowing posteriorly to the ribs. This was punctured with a hypodermic needle, and the material evacuated was examined by Professor Jeffries, together with the discharge from the urethra and prostate gland, but no evidences of the gonococcus or tubercle bacillus were found. About ten days ago another fluctuating tumor began to develop on the right hand, about one-half an inch posterior to the first joint of the little finger. The patient has suffered no pain from any of these lesions. The treatment has been oil of gaultheria locally applied and given internally, without benefit; iodide of potash has also been given internally, without, however, reducing any of the swellings. The joint lesions are still swollen and discharge a greenish material, the abscess on the chest wall has not yet closed, and the chronic urethritis is still present.

Dr. F. C. Keller said that from the history and general appearance of the patient, he was inclined to think the lesions of tubercular origin. He had seen several cases of tubercular arthritis, and this case had many features in common with them.

Dr. V. C. Pedersen said he had seen two or three cases of gonorrheal arthritis, but the patients all suffered severe pain, and there was no destruction of bone, but some destruction of the joint tissue. He had read of one case of gonorrheal infection where the patient died and pure gonococci were recovered from the fluid in the pericardium and in the pleura after death. The patient under discussion presented some general appearance of gonorrheal arthritis, but the slow onset of the joint symptoms, their comparative mildness, their wide distribution and the tuberculous temperature and sinus formation, and the difficulty in obtaining any pathological findings from the fluid in the joints, all seemed, in the speaker's opinion, to point to tuberculosis.

Dr. A. Lyle said he thought the condition tuberculous. The destructive process in the joints, together with the lack of gonococci in the discharge seemed to prove rather conclusively that it was of a tuberculous nature.

Dr. Jeffries said he had examined the fluid on two occasions, and, finally, the fluid from the cavity of the chest, and had been unable to find gonococci of any sort, by examination or culture. He could only assume that the condition was tubercular, because, in the absence of any demonstrable form of germ in pus we can only come to this conclusion, although the pus does not necessarily contain tubercle bacilli. If the walls of the abscess were scraped, some tubercle bacilli might be obtained, or the histological structures of tuberculosis may be demonstrated. The bacilli, by the time they have become part of the pus, have so far undergone disintegral changes that they fail to accept the dyes, and thus there is pus without any demonstrable bacilli.

Dr. M. Franklin said he had examined the patient with the fluoroscope, and the appearance of the bones was unquestionably tubercular. The fluoroscope, however, unaided by the X-ray photograph, was far from reliable.

Dr. James Pedersen said that possibly the patient had had a more or less pure gonococcal arthritis of the elbow joint; but that in his opinion the sinus formation about the finger-joints and in the testicle were significant of a tuberculous process there. He had never seen a gonococcal infection of joints behave just as these finger-joints were behaving, and the history of the case, together with the clinical picture, and the pathologic findings together with the fluoroscopic picture, as already stated, seemed to him conclusive.

Dr. M. Packard said the joints of the patient's fingers reminded him of the picture of spinoventosa, which occurs in specific and tubercular patients. A positive diagnosis might be made by injecting some of the fluid from the patient's abscess into a guinea-pig.

Dr. L. L. Roos said that the apex of the right lung showed some fine rales and a slight dullness, which was indicative of tuberculosis.

### PATIENT SUFFERING FROM FACIAL PARALYSIS.

Dr. G. B. McAuliffe presented a patient who, during the course of an acute otitis media, developed facial paralysis. When this occurs as the result of the otitis, it is due to the pressure of an exudate on the nerve exposed by reason of a bony dehiscence or due to a neuritis established by inflammatory extension through the small foramina in the facial canal. The case was referred to Dr. Franklin for electrical treatment.

### FIBROMA OF INSIDE CHEEK.

He also showed a patient with a small fibroma inside of the cheek, which had existed for twenty years, and which got so continually between the teeth that the woman had developed the habit of speaking with the jaws almost set. The wonder is that she endured it so long.

EPITHELIOMA OF THE LOWER LIP.

Dr. J. A. Bodine showed a case of epithelioma of the lower lip on which he had operated. The patient had been treated for three months under the X-rays, with a resultant increase in size of the growth. He first appeared at the clinic with a foul, fungus mass, which involved the lower lip from the angle to within a third of an inch of the other angle, and extended downward over the chin.

The speaker said he never put a dressing on a face wound. He had removed upper and lower jaws and lower lips, and closed hare lips, and had never applied any gauze dressing, and yet never had any suppuration. The reason for not applying a dressing is that if it be under the eye, the dressing becomes saturated with tears; and if below the nostrils, the nasal secretion will infect the wound, or below the mouth the saliva will be retained and decomposed in the gauze, thus infecting a wound that would otherwise heal. In the operation under discussion, every single gland and every bit of loose aveolar tissue was dissected down to the clavicle and both submaxillary glands were removed, care being taken not to sever the facial artery, as the life of the flaps depends upon the integrity of this artery.

Dr. Franklin said that he had never known of an epithelioma of the lower lip being cured by exposure to X-rays, and did not believe that it could be done. He thought that now, however, was the time to submit this patient to X-ray treatment, and thought that about fifteen radiations would materially lessen the chances of recurrence.

Dr. D. S. Dougherty said that he would like to send his mastoid patients around with a very light dressing, and was much impressed with Dr. Bodine's idea of getting patients with face wounds out of bed in a short time. He always got his mastoid patients up, if possible, on the second day, and even allowed them to leave the hospital two or three days after operation.

POTTS'S DISEASE OF THE DORSAL SPINE.

Dr. V. C. Pedersen presented this patient, three months after operation, in order to illustrate the importance of stripping a patient for physical examination. The abscess in the case had pointed at the tip of the left twelfth rib, where a small incision had been made before the case came into Dr. Pedersen's hands. No examination of the back had been made, so that the kyphosis present in the mid-dorsal region escaped attention. At first the cavity drained freely, but under the persistent use of 10 per cent. iodoform in glycerine emulsion, all discharge had ceased.

With the aid of orthopedic corsets, prescribed by Dr. Homer Gibney, further progress of the disease appeared to have been arrested. The usual systemic treatment for tuberculosis was also being followed. Dr. Pedersen stated that he hoped to have the boy leave the city, and take up farming as his life's work.

PERIURETHRAL ABSCESS.

Dr. Pedersen also presented two cases of periurethral abscess. The first patient developed complications before any treatment of the disease was had, in the midpenile region. The speaker adopted the plan of treatment which he thinks is best in these cases—not only opening the abscess throughout its entire length in the long axis of the penis, but also of slitting the skin somewhat in addition, thus producing a large cone-shaped cavity, with its apex at the urethra, and its base in the skin sheath of the penis, thus facilitating packing from the bottom. In this case the entire cavity was closed in three weeks. At no time previous to or subsequent to the operation was there a communication between the abscess and the urethra. The expectant method of treating the gonorrhea had resulted in a perfectly healed urethra in the course of twelve weeks' treatment, which included the surgical care of the abscess.

The second case was one of periurethral abscess in the glans penis, which appeared during the course of a gonorrhea, but without material symptoms. When first seen by Dr. Pedersen, the gonorrhea was well, with the exception of a few shreds in the first urine passed. The abscess was about the size of a pea, and entirely without subjective symptoms. Pressure did not show any connection with the cavity of the urethra. A hypodermatic needle was passed into the abscess and withdrew a few drops of mucopus, which, unfortunately, were lost in the dispensary before examination for gonococci could be made. Since that time the hypodermatic needle-hole in the wall of the abscess has remained open, but no material discharge which the patient noticed was coming away, and no urine escaped. As to treatment, the speaker thought that the proper method was to open the region about the abscess as far as the floor of the urethra, and tie off the neck of the abscess at this point, and then dissect out the wall, allowing the wound to heal by granulation. He thought this method would result in complete and steady cure. Tne case was presented as showing how an abscess in the fosso navicularis could develop with few symptoms, subjective or objective.

Dr. James Pedersen opened the discussion of these cases. He had never seen an exact duplicate of the second case presented, that of abscess in the glans near the frenum. Abscesses in that locality were much more frequent on one side or the other of the frenum. If the abscess in question was opened too freely, he thought there was risk of a sinus through which urine would leak, and which would be difficult to close because of the scant amount of tissue with which to do a plastic operation. The glans penis does not lend itself well to plastic work. He thought the abscess might be punctured with a very slender bistoury, from within the meatus.

The second case, that of the periurethral abscess near the penoscrotal angle, he thought had been very skillfully treated. A maneuver he had seen practiced in these cases consisted in making the usual free incision in the skin, over the prominence of the tumor, but in incising the abscess cavity either above or below the center, so that, in case the floor of the urethra sloughed at the site of the abscess, the resulting sinus would have an oblique direction, and could therefore be the more easily closed, with or without a plastic operation.

Dr. D. A. Sinclair said that in cases of periurethral abscess, he always tried to have the discharge into the urethra, rather than external, even though the skin was slightly yellowish, showing that the abscess wall was about to burst, because by incision into the urethra, danger of fistula is removed. The incision is made by passing an endoscopic tube into the urethra, making a free incision into the abscess, and then using a twenty-five per cent. solution of peroxide of hydrogen once a day, through the urethral opening. When the abscess bursts externally, and a fistulous tract results, the sinus is closed by dissecting around it from the skin surface to the urethra, and tying a ligature around it just below the mucous membrane, with the result of nearly always closing it.

Dr. Lyle said he did not think anything was being accomplished by the iodoform drain. He had been using recently a solution of iodine, and irrigating with it from below the sinus, and in one case, particularly, had obtained very good results after five irrigations of five per cent. iodine. A tuberculous abscess was opened at the clinic, and three pints of pus removed, and it was curetted and washed out with iodine once a week for five weeks, with excellent results.

Dr. Bodine said that he would have known Dr. Pedersen was a general surgeon by the scar of the periurethral abscess case. He advocated draining these abscesses externally, rather than into the urethra, as the latter process does not follow the rule of general surgery of dependent drainage.

Dr. V. C. Pedersen said, in answer to a query from Dr. J. Pedersen, that the little pocket of pus is situated well in front of the frenum in the glans penis proper. This came during an attack of gonorrhea, but without symptoms of pain, and he thought it some congenital condition, which had developed later. He thought a rather free opening and tying off the same at the floor of the urethra, the correct one, and also agreed with Dr. Bodine that an abscess containing pus, whether prostatic or urethral, should be dealt with on surgical principles. An ischiorectal abscess is not drained from the canal of the rectum, but from a skin opening liberal enough to guarantee drainage from the very bottom. After opening the abscess to its utmost limitations, extra cuts should be put in the skin, so that the skin will not heal first. He had tried the iodine irrigations on other patients, with very good results. Lugol's solution of iodine, he thought, was better than the simple solution, as the former prevented the precipitation of the iodine and enabled it to work its way through the sinuses.

Dr. Frederick E. Beal read the paper of the evening, which was entitled:

### THE RECOGNITION AND DIFFERENTIATION OF RALES.

He said, in part:

"In my opinion, the rales heard in the lung should be divided into but two main divisions, dry and moist; and that it is as easily possible to differentiate between a dry and a moist rale as it is to tell the difference between the sound of a piano and that of a violin. It is true that a dry rale may vary in its size from that produced by the largest bronchus to one having its origin in the tiniest bronchial; while the size of the moist rale may be that of the finest air-vesicle to that of the largest cavity, and the number of rales, each of different size, that could possibly exist between these extremes is almost without limit. Why, then, should we confuse ourselves by picking out two or three of each kind, and giving these the dignity of a name? It is enough to know, and is of the greatest importance, from a diagnostic and prognostic standpoint, that a rale is moist or dry; and this can with certainty be known, while its size, though important, can be told with a moderate degree of accuracy, by its pitch.

"The greatest point of differentiation is that a dry rale has always some duration to it, while a moist rale is always instantaneous. A dry rale is caused by air going at a given rate of speed through a smaller sized tube than it normally should do, thereby raising the pitch of the sound this passage of air produces. The commoner causes of dry rales are mucus stuck so tenaciously on the side of the bronchus that breathing does not move it; this will lessen the calibre of that bronchus. Any pressure from without, or any foreign body or growth within, that would encroach upon the size of the air-passage, and, finally, any nervous phenomenon that would spasmodically cause the bronchi to lessen their calibre, would all give rise to the same condition.

"The moist rale is caused, either by the pulling apart of moist adhered surfaces, or by air passing through an accumulation of fluid that is sufficiently liquid to allow it to bubble through. This size may be caused by the breaking apart of the stuck-together surfaces of the finest air-vesicle, or that of the pulling apart of the walls of a collapsible cavity, or the air may break through a collection of thin mucous filling a bronchus; or, again, a cavity may be more or less filled with fluid, and entered below the surface of that fluid by a bronchus. The air entering through the bronchus will bubble up through the liquid, but the sound produced will always be instantaneous.

"The moist rales are of far graver significance than the dry. The dry rales are the whistlings heard, perhaps, in their greatest profusion and purity in bronchitis, emphyza and asthma, while the moist rales are most typical in pneumonia and tubercular pulmonalis."

Dr. V. C. Pedersen asked where the reader placed the friction rale of pleurisy.

Dr. Beal said that the dry rale, or the rale with duration, always sounds a little at a distance when listening to it, and the moist rale always sounds closer to the ear. The difference between these and the friction rale of pleurisy is that, while the friction rale has duration, it always sounds close to the ear. It is sometimes confounded with the dry rale.

---

A western congressman whose testimonial of a patent medicine has of late appeared in all the papers, has recently received a remarkable letter from a person who evidently thinks the congressman has the remedy on sale as a sort of congressional side line. The letter follows :

"Dear friend & Statesman : I rite you the urliest dait to be so cind as to do me a fafor. I haf trid all cinds of patent medisin for heart decetse an no avail. I red your little pome on Hart deces beginnin

" 'The hart which sad tumultus beets,
with throbs of keenest pain
wil oft recover its defects
Thro' natur's sweet refrain.'

"I now ask you to send me by return male 2 bottles of your medsin naturs sweet refrane. I haf never trid an injun doc but haf took all cinds erbs. Sen to ——— ——— ——— Penn.

"P. S.—I will sen prise by return male."

